# Global longitudinal strain and echocardiographic parameters of left ventricular geometry and systolic function in healthy adult Angolans: Effect of age and gender

**DOI:** 10.21542/gcsp.2022.2

**Published:** 2022-06-30

**Authors:** Humberto Morais, Ana Feijão, Savarino de Victória Pereira

**Affiliations:** 1 Department of Cardiology. Hospital Militar Principal/Instituto Superior, Luanda, Angola; 2 Department of Cardiology. Luanda Medical Center, Luanda, Angola; 3 Service of Cardiology. Hospital Américo Boavida, Angola

## Abstract

Introduction: Studies on the normal echocardiographic reference values in Africans are limited.

Objectives: This study aims to establish the normal left ventricular echocardiographic parameters for adult Angolans, stratified by gender and age.

Methods: A cross-sectional study was performed involving healthy adults attending a diagnostic center in Luanda, Angola. The two-dimensional transthoracic echocardiography was performed according to the Guidelines of the American Society of Echocardiography and the European Association of Cardiovascular Imaging.

Results: A total of 103 men (47.5%) (mean age: 39.5 ± 10,8) and 114 women (52.5%) (mean age: 43.0 ± 12,5 years) were included. Compared to men, women were older (*p* = 0.025) and had a significantly smaller body surface area (BSA) (*p* < 0.001). Left ventricular wall thickness, left ventricular end-diastolic dimension and volume (LVEDV), left ventricular mass (LVM) and LVM indexed to BSA (LVMi) were significantly lower in women (*p* < 0.005). LVEDD indexed to BSA and left ventricular ejection fraction (LVEF) were significantly higher in women than in men (*p* = 0.007), (*p* = 0.01), respectively. Mitral annular plane systolic excursion, LVEF by strain, and global longitudinal strain showed no gender differences. Posterior wall thickness showed a statistically significant increase in the older groups (*p* = 0.043). The VST, relative wall thickness, LVM, and LVMi showed no significant differences between age categories. In turn, the shortening fraction and the ejection fraction increase with age.

Conclusion: For a more sensitive morphological and functional assessment of the left ventricle, it is necessary to take into account the gender and age of the individual.

## Introduction

Echocardiography is the imaging method most often used to assess anatomy and cardiac function worldwide. Many studies have focused on the influence of age, sex, and race/ethnicity/nationality on echocardiographic parameters in healthy populations^1–5^. Several authors have questioned whether the parameters referred to in the ASE and ESC guidelines should serve as reference values for all populations, since they are derived from studies conducted on Americans and Europeans. Some researchers suggest that studies should be conducted to determine the reference echocardiographic values for their population ^3,6,7^. With increased awareness of the importance of accounting for age, sex, and ethnicity, several studies have obtained normal reference ranges for echocardiography and Doppler data for specific healthy populations^8^.

Moreover, in a study published in 2019 by Ash et al., and carried out in 15 countries that included 2008 healthy individuals, the same methodology was used in all centers. The authors concluded that the dimensions and volumes of the left ventricle (LV) were larger in men, while the ejection fraction and values of the global longitudinal strain (GLS) were higher in women. Inter-country variability is significant for left ventricular volumes; therefore, nationality should be considered when defining the ranges of normality^9^.

The present study is the second part of a pilot study on echocardiographic values of left ventricular (LV) systolic and diastolic function in healthy adult Angolans. The first part focused on LV diastolic function and was published previously (10). This second part aims to evaluate the left ventricular end-diastolic dimensions and volumes, LV systolic function, and left ventricular global longitudinal strain (GLS) using transthoracic echocardiography in healthy adult Angolans. In addition, the effects of aging and sex on these parameters were evaluated.

## Material and Methods

This methodology has been described in detail elsewhere^10^. Briefly, a cross-sectional descriptive observational study was carried out in a single diagnostic center in Luanda, Angola. We recruited individuals who were referred to our echocardiography laboratory. The study included 217 healthy individuals aged 18 years or older who fulfilled the inclusion criteria and agreed to participate in the study. Age, sex, weight, height, and the presence or absence of cardiovascular risk factors (CVRF) were recorded on the day of echocardiography. The body surface area (BSA) was calculated using the formula BSA = 0.007184 × [(height (cm)] ^0.725^ × [(weight (kg)] ^0.425^ andbody mass index (BMI) was calculated by the formula BMI = weight (kg)/height^2^ (cm). Systolic blood pressure (SBP) and diastolic blood pressure (DBP) were measured by a specialized healthcare professional minutes before the echocardiographic examination using an aneroid sphygmomanometer, according to the Korotkoff method (k1 to k5 sounds, respectively).

### Two-dimensional transthoracic echocardiography

Echocardiography was performed using commercially available equipment (Mindray DC-70 exp Diagnostic Ultrasound System with a P4-2 transducer). Two-dimensional transthoracic echocardiography (TTE) was performed and analyzed according to the Guidelines of the American Society of Echocardiography and the European Association of Cardiovascular Imaging^11^ by a single experienced and accredited echocardiographer.

The ventricular septal thickness (VST), posterior wall thickness (PWT), and diastolic diameter of the left ventricle (LVDD) were measured in end-diastole, and the left ventricle systolic diameter (LVSD) was measured in end-systole. All measurements were performed in M-mode and guided by two-dimensional echocardiography. LVM was calculated using the following formula: LVM = 0.8 × {1.04 [(LVEDD + PWT+IVT) 3 − (LVEDD) 3]) + 0.6^10^. The LVM index (LVMI) was calculated using the formula LVMI = LVM/BSA^10^. RWT was calculated according to the following formula: RWT= 2xPWT/LVEDD^10^. Left ventricular shortening fraction (LVSF) was calculated according to the following formula: LVEDD-LVESD/LVEDD*100. Left ventricular ejection fraction (LVEF) was calculated using the Simpson method. Left ventricular systolic function was classified as preserved if LVEF was ≥55%^11^.

### Global left ventricular longitudinal strain

High-quality images of the long-axis, four-chamber, and two-chamber views were acquired to evaluate the global longitudinal strain (GLS) parameters of the left ventricle. The GLS values were obtained according to a previously reported method^12^.

### Inclusion and exclusion criteria

The study included healthy individuals aged 18 years or older who did not have exclusion criteria and agreed to participate in the study. A questionnaire was given to individuals about possible known diseases (cardiac and non-cardiac). Individuals with chronic conditions or chronic medications were excluded. Pregnant women, high competition athletes, and individuals with heavy alcohol consumption were excluded. Arterial hypertension was ruled out based on the measurement taken before the examination; individuals with SBP ≥140 mmHg and/or DBP ≥90 mmHg, and individuals with BMI ≥40 kg/m^2^ were excluded. Individuals with abnormal values of cholesterol, triglycerides, creatinine, or glucose found in their medical records in the last six months were also excluded. Left ventricular ejection fraction mild) valvular heart disease or pericardial disease were also exclusion criteria.

### Statistical analysis

Data were analyzed according to sex and age group stratification. The normality of distribution was analyzed using the Shapiro–Wilk test in samples with a size less than 30. In samples with a size greater than 30, the normality of distribution of values was accepted according to the central limit theorem. Qualitative variables were expressed by absolute and relative frequencies and quantitative variables with means and standard deviations. Statistical significance was set to <.05. Independent-samples *T*-test, one-way ANOVA, and Chi-square independence were used. The lower and upper normal limits of the conventional echocardiographic parameters were established as the mean ±2SD measurement. Data were analyzed using SPSS 27.0 for Windows.

## Results

A total of 103 men (47.5%) and 114 women (52.5%) (mean age: 41.3 ± 18,1 years) were included. There were no significant gender differences in BMI, SBP, and DBP. However, women were older (43 years versus 39 years, *p* = 0.025) and showed significantly smaller BSA (1.8 versus 1.9; *p* < 0.001) compared to men.

Table 1 shows the echocardiographic parameters of left ventricular geometry and systolic function in the entire population and according to gender. Compared to women, men had larger left ventricular dimensions (48.1 vs 45,8, *p* < 0.001) and volumes (101.0 vs 91.7, *p* < 0.001), had higher VST (9.6 vs 9.0 *p* < 0.001), higher PWT (9.5 vs 8.6; *p* < 0.001) and LVM (162.1 vs 133.9; *p* < 0.001). When LVM was indexed for BSA or height, it remained statistically higher in men than in women.

**Table 1:** Echocardiography parameters in the entire population by sex.

	Total (217)	Male (103)	Female (114)	*p* Value.
	Mean ±SD	Mean ±SD	Mean ±SD	
VST (mm)	9.3 ± 1.4	9.6 ± 1.3	9.0 ± 1.5	0.001^***^
PWT (mm)	9.0 ± 1.4	9.5 ± 1.2	8.6 ± 1.4	0.001^***^
LVEDD (mm)	46.9 ± 4.7	48.1 ± 4.4	45.8 ± 4.7	0.001^***^
LVEDD/BSA (mm/m^2^)	25.6 ± 2.8	25.1 ± 2.5	26.1 ± 3.1	0.007^**^
LVEDV (ml)	96.1 ± 21.2	101.0 ± 23.2	91.7 ± 18.3	0.001^***^
LVEDVBSA (ml/m^2^)	52.2 ± 10.6	52.4 ± 11.2	52.1 ± 10.1	0.832
RWT	0.39 ± 0.07	0.38 ± 0.07	0.40 ± 0.06	0.020^*^
LVM (g)	147.3 ± 39.5	162.1 ± 35.2	133.9 ± 38.5	0.001^***^
LVM/BSA (g/m^2^)	80.0 ± 20.0	84.3 ± 17.4	76.0 ± 21.5	0.002^*^
LVM/height (g/m)	86.9 ± 22.6	92.8 ± 19.9	81.6 ± 23.7	.001^***^
LVSF (%)	40.1 ± 7.5	39.2 ± 7.1	40.8 ± 7.7	0.115
LVEF (%)	69.6 ± 8.2	68.2 ± 8.4	71.0 ± 7.7	0.010^**^
MAPSE (mm)	15.0 ± 2.7	15.1 ± 2.7	15.0 ± 2.6	0.841
FEVE/Strain (%)	52.3 ± 5.6	52.2 ± 5.8	52.3 ± 5.4	0.910
GLS (%)	18.3 ± 2.8	18.0 ± 2.7	18.5 ± 2.9	0.226

**Notes.**

BSA body surface area GLS global longitudinal strain LVEDD left ventricular end-diastolic diameter LVEDV left ventricular end-diastolic volume LVEF left ventricular ejection fraction LVM left ventricular masse LVSF left ventricular shortening fraction MAPSE mitral annular plane systolic excursion PWT posterior wall thickness RWT relative wall thickness SD Standard Deviation VST ventricular wall thickness

* *p* ≤ 0.05.

** *p* ≤ 0.01.

*** *p* ≤ .001.

**Table 2:** Baseline clinical characteristics in the entire population by age.

	Total (217)	18–29 (36)	30–39 (71)	40–49 (53)	50–59 (40)	≥ 60 (17)	*P* Value.
	Mean ±SD	Mean ±SD	Mean ±SD	Mean ±SD	Mean ±SD	Mean ±SD	
M/F(%)	47.5/52.5	55.6 ±/44.4	56.3/43.7	39.6/60.4	16/24	35.3/64.7	0.171
BSA (m^2^)	1.8 ± 0.2	1.8 ±.1a	1.9 ± 0.2 b	1.9 ± 0.2	1.8 ± 0.2	1.8 ± 0.1	0.004^**^
BMI (Kg/m^2^)	25.8 ± 4.2	23.6 ± 3.7a	26.1 ± 4.4	26.2 ± 4.1	26.7 ± 4.1b	26.6 ± 3.5b	0.008^**^
SBP (mmHg)	126.5 ± 9.7	118.1 ± 12.9a	127.0 ± 8.1b	127.0 ± 8.3b	130.0 ± 7.3b	132.2 ± 5.8b	0.001^***^
DBP (mmHg)	73.5 ± 8.3	68.1 ± 8.5a	73.7 ± 7.7b	73.6 ± 7.8b	75.2 ± 7.9bc	79.5 ± 6.7c	0.001^***^

**Notes.**

BSA body surface area BMI body mass index DBP diastolic blood pressure F female M Male SD Standard Deviation SBP systolic blood pressure

* *p* ≤ 0.05.

** *p* ≤ 0.01.

*** *p* ≤ .001.

There is a significant statistically difference between the groups with the letters a and b; a and c; b and c.

**Table 3:** Echocardiographic parameters in the entire population by age.

	Total	18–29 (36)	30–39 (71)	40–49 (53)	50–59 (40)	≥ 60 (17)	*p* Value
	Mean ±SD	Mean ±SD	Mean ±SD	Mean ±SD	Mean ±SD	Mean ±SD	
VST (mm)	9.3 ± 1.4	9.1 ± 1.4	8.9 ± 1.4	9.5 ± 1.2	9.6 ± 1.7	9.7 ± 1.3	0.056
PWT (mm)	9.0 ± 1.4	8.6 ± 1.3a	8.9 ± 1.5	9.0 ± 1.2	9.4 ± 1.4	9.5 ± 1.3b	0.043^*^
LVEDD (mm)	46.94.7	46.2 ± 3.8	47.5 ± 4.9	46.5 ± 5.3	46.9 ± 4.8	47.3 ± 3.8	0.630
LVEDD/BSA (mm/m^2^)	25.6 ± 2.8	26.3 ± 2.1	25.3 ± 2.6	25.2 ± 3.0	25.6 ± 3.4	26.8 ± 3.3	0.139
LVEDV (ml)	96.1 ± 21.2	90.3 ± 20.0	99.7 ± 23.7	98.4 ± 19.8	91.6 ± 17.9	96.8 ± 21.8	0.125
LVEDV/BSA (ml/m^2^)	52.2 ± 10.6	51.2 ± 10.1	52.8 ± 11.2	53.2 ± 9.3	50.0 ± 10.4	54.7 ± 13.0	0.449
RWT	0.39 ± 0.07	0.37 ± 0.05	0.38 ± 0.07	0.39 ± 0.07	0.41 ± 0.07	0.41 ± 0.07	0.109
LVM (g)	147 ± 39	137 ± 35	145 ± 39	147 ± 39	155 ± 46	158 ± 31	0.235
LVM/BSA (g/m^2^)	80.0 ± 20.0	77.6 ± 17.2	76.8 ± 18.6	79.2 ± 17.2	84.6 ± 25.9	89.9 ± 20.6	0.069
LVM/height (g/m)	86.9 ± 22.6	81.2 ± 19.9	84.3 ± 21.1	87.0 ± 21.8	92.7 ± 28.3	96.1 ± 18.2	0.071
LVSF (%)	40.1 ± 7.5	37.5 ± 6.0 a	39.5 ± 7.6 a	40.7 ± 6.9 a	40.4 ± 6.9	45.3 ± 10.0 b	0.008^**^
LVEF (%)	69.6 ± 8.2	68.1 ± 7.9 a	68.0 ± 8.8 a	70.6 ± 7.7	70.8 ± 6.8	74.2 ± 8.9 b	0.024^*^
MAPSE (mm)	15.0 ± 2.7	15.1 ± 2.5	15.1 ± 2.4	15.1 ± 2.4	14.6 ± 3.1	15.5 ± 3.6	0.829
LVEF/Strain (%)	52.3 ± 5.6	54.0 ± 5.6	52.0 ± 6.2	52.0 ± 4.5	51.3 ± 5.9	53.0 ± 5.0	0.275
GLS (%)	−18.3 ± −2.8	−18.8 ± −2.2	−18.4 ± −3.0	−18.1 ± −2.6	−17.8 ±−3.2	−18.6 ±−2.8	0.534

**Notes.**

BSA body surface area GLS global longitudinal strain LVEDD left ventricular end-diastolic diameter LVEDV left ventricular end-diastolic volume LVEF left ventricular ejection fraction LVM left ventricular masse LVSF left ventricular shortening fraction MAPSE–PWT posterior wall thickness RWT relative wall thickness SD Standard Deviation VST ventricular wall thickness

* *p* ≤ 0.05.

** *p* ≤ 0.01.

*** *p* ≤ .001.

There is a significant statistically difference between the groups with the letters a and b.

Regarding the parameters of systolic function quantification, only LVEF was statistically different between sexes, showing a higher value in women than in men (71% vs. 68%; *p* = 0.01). Women also had a greater LVSF (41% vs. 39%, *p* = 0.115). The remaining parameters, such as mitral annular plane systolic excursion (MAPSE), LVEF by strain, and GLS, showed no differences between sexes.

Table 2 summarizes the demographic data of the entire population according to the age categories. Compared with individuals younger than 30 years, all the remaining four age categories had significantly higher SBP and DBP. Patients aged >49 years also had a higher BMI than those aged <30 years. Patients in the 50–59 years age group had lower DBP than those older than 59 years.

Table 3 shows the parameters of left ventricular geometry and systolic function in the entire population and according to age category. Regarding morphological parameters, the PWT values showed a statistically significant increase in the older groups (*p* = 0.043). Although there was an increase in IVS, RWT, LVM, and LVM indexed to BSA and height with increasing age, none of these parameters demonstrated a significant difference between the various age groups.

Regarding the parameters for quantifying systolic function, LVSF and LVEF values significantly increased with age (*p* = 0.008 and *p* = 0.024, respectively). In turn, the GLS decreased with age (*p* = 0.603).

Table 4 shows the lower and upper normal limits of the echocardiographic parameters for the present study population by sex and its comparison with the 2015 ASE/EACVI guidelines. Our results show that the upper normal limits (UNL) for the LVWT and the left ventricular mass in both, males and females, and the left ventricular end-diastolic diameters and volumes in women were slightly higher than those proposed by the 2015 guidelines. In contrast, in our study, the low normal limits (LNL) for LV GLS were considerably lower than those suggested by the 2015 guidelines.

**Table 4:** Lower and upper normal limits of the echocardiographic parameters for the present study population by sex and its comparison with 2015 ASE/EACVI guidelines.

	Present study, LNL to UNL			Guidelines 2015	
	Male	Female	P Value	Male	Female
VST (mm)	7 to 12	6 to 12	.001^***^	6 to 10	6 to 9
PWT (mm)	7 to 12	6 to 11	.001^***^	6 to 10	6 to 9
LVEDD (mm)	39 to 57	36 to 55	.001^***^	42 to 58	38 to 52
LVEDD/BSA (mm/m^2^)	20 to 30	20 to 32	.007^**^	22 to 30	23 to 31
LVEDV (ml)	55 to 147	55 to 128	.001^***^	62 to 150	46 to 106
LVEDV/BSA (ml/m^2^)	30 to 75	32 to 72	.832	34 to 74	29 to 61
RWT	0.20 to 0.52	0.30 to 0.50	.020^*^	NA	NA
LVM (g)	92 to 232	57 to 211	.001^***^	88 to 224	67 to 162
LVM/BSA (g/m^2^)	49 to 119	33 to 119	.002^*^	49 to 115	43 to 95
LVM/height (g/m)	53 to 133	34 to 129	.001^***^	NA	NA
LVSF (%)	25 to 53	25 to 56	.115	NA	NA
LVEF (%)	51 to 85	56 to 86	.010^**^	52 to 72	54 to 54
MAPSE (mm)	10 to 20	10 to 20	.841	NA	NA
FEVE/Strain (%)	41 to 64	41 to 63	.910	NA	NA
GLS (%)	−13 to 23	−13 to 24	.226	NA	NA

**Notes.**

BSA body surface area GLS global longitudinal strain LVEDD left ventricular end-diastolic diameter LVEDV left ventricular end-diastolic volume LVEF left ventricular ejection fraction LVM left ventricular masse LVSF left ventricular shortening fraction LNL Low normal limit MAPSE–PWT posterior wall thickness RWT relative wall thickness SD Standard Deviation UNL Upper normal limit VST ventricular wall thickness

* *p* ≤ 0.05.

** *p* ≤ 0.01.

*** *p* ≤ .001.

LNL and UNL were established as the mean ±2SD.

## Discussions

In this study, LV cardiac structure, LV systolic function, and LV global longitudinal strain parameters were assessed by echocardiography in a cross-sectional sample of healthy adult Angolans. 

### Differences between sexes

The main findings of our study showed that LVWT, LVEDD, LVEDV, LVM, and LVM indexed to height and BSA were significantly lower in women (*p* < 0.005). Our results are similar to those reported in studies performed in other populations, including Brazil^1^, Iran^4^, Europe^13^, sub-Saharan Africa^14^, Korea^15^, and Turkey^16^.

However, the present study showed that LVEDV indexed to BSA was not statistically significant (*p* = 0.832) between sexes. In turn, the LVEDD indexed to BSA was statistically significantly higher in women compared to men (*p* = 0.007). Interestingly, in a study conducted in Iran, the authors also found that LVEDD indexed to BSA was higher in women than in men^4^.

Regarding left ventricular function, our study showed that both LVSF and LVEF were higher in women than in men. However, only LVEF showed a statistically significant difference between sexes (*p* = 0.01). Our results are consistent with those reported by Asch et al. and Nel et al.,^9,14^. On the other hand, Angelo et al. also reported a statistically significant difference between sexes in LVSF, but not in LVEF^1^. Our study does not show any difference between genders in GLS (18.5 versus 18.0, *p* = 0.190) which contrasts with the one reported by Sullere et al., Lang et al. and Ash et al., which state that there is evidence that women have slightly higher GSL values compared to men^5,9,11^.

### Age-related differences

Concerning age-related differences, Our study showed that only PWT increased significantly with age. Although VST, LVEDD, and LVM increased with age, the difference was not statistically significant. In contrast, our study showed that LVSF and LVEF increased significantly with age. On the contrary, MAPSE, LVEF strain, and GLS all show a reduction with age, although they have no statistical significance.

Regarding the data on the influence of age on LV morphological parameters and LV systolic function, the existing studies are controversial. Ventricle wall thickness and LVM are morphological parameters that are highly dependent on loading conditions that change physiologically with age, such as arterial blood pressure. Several authors reported an increase in wall thickness and LV mass with age^2–4,15,17^, while others, despite presenting an increase in left ventricular mass, did not present statistical significance^1,14^. Our study showed that only PWT increased significantly with age.

Some studies have reported that LVEDD and LVEDV decrease with aging^1–3,13,14^. On contrary, and in line with another study, we did not find a significant change in LVEDD and LVEDV with age^15^.

Left ventricular ejection fraction is the echocardiographic parameter with the greatest influence on clinical and therapeutic decisions, and the importance of perceiving its behavior with age is imperative. The effects of aging on LV systolic function reported in several studies have been controversial. Some authors have reported no change in LVEF^1,2,14^, while others have reported a decrease in LV systolic function^4,18^. In line with other studies^3,13,19^, our results also showed an increase in LVSF and LVEF with age.

The limitations of systolic parameters such as LVSF and LVEF are well known, and LV GLS emerges as an innovative technique that allows quantification of sub-clinical changes in LV systolic function, being a technique parameter that does not depend on the load conditions and is capable of presenting subtle changes LV systolic function. Although not statistically significantly, our study showed that the GLS decreased with age, which is consistent with previously reported results^5,10,19^.

### Our results *versus* the normal reference values recommended in 2015 Guidelines

The recent American Society of Echocardiography (ASE) and European Association of Cardiovascular Imaging (EACVI) updated recommendations for chamber quantification to define ranges of normative values for the general population using data obtained from well-designed population-based studies^11^. Although these normative values are used as a reference worldwide, they are derived from data obtained in the United States or specific regions in Europe, thereby reflecting a predominantly white population that is not representative of patients from other races or areas of the world^9^. Reports from Japan^2^, China^3^, Iran^4^, and India^20^ suggest that “normal” hearts in these nations are smaller than those reported in American and European studies.

Furthermore, a study published by Qureshi et al. showed large observed differences in reference limits for Hispanics/Latinos compared to ASE chamber quantification guidelines^5^. The authors found that both 2005 and 2015 suggested cut-off guidelines underestimated the measures of LVM, VST, and RWT.

In contrast, these thresholds overestimated the LVEDV, LVEDD, and PWT in both men and women. These observations depict relatively thicker and smaller healthy hearts in individuals of Hispanic/Latino origin compared with ASE guideline-defined reference values.

In line with the results reported by Qureshi et al., our study also showed that in our population, the values of LVWT and left ventricular mass in both men and women were higher than those proposed in the 2015 guidelines^5^. On the other hand, our results showed that the values for left ventricular diameters and volumes in men are very similar to those proposed in the 2015 guidelines, while in women these values are higher than those proposed in the guidelines, even when they are indexed to BSA, which is in agreement with the results of the WASE Normal Values Study^9^.

The 2015 guidelines did not provide normal ranges for LV GLS, but only suggested a consensus-based abnormality cutoff value of −20%. The current study provides the lower normal limits for GLS, which are considerably lower (-13% for men and-13% for women) than those found in the WASE Normal Values Study (−17% and −18%, respectively)^9^. However, these data must be interpreted with caution, as the strain analysis to date is vendor-dependent.

Study limitations: Subclinical disease was not excluded because hemoglobin, thyroid hormone, glucose, and lipid levels were not assessed. We considered the exclusion criteria to include only healthy adults. However, more complex tests, such as impaired glucose tolerance, cardiac catheterization, or diastolic stress echocardiography, were not performed to unmask subclinical diastolic dysfunction and symptoms. The sample size may not be sufficient to extrapolate the data presented herein to the Angolan population, but it certainly serves as a reference for future work in this area. The sample size of healthy participants aged 60 years or older was small. One of the limitations was that a single echocardiographer in the department performed all the echocardiograms.

## Conclusion

For a more sensitive and accurate morphological and functional assessment of the left ventricle, it is necessary to consider the sex and age of the individual.

### Author statement

Study design: HM and AF

Data collection: AF

Writing of the manuscript: HM, AF and SVP

Revisions and approval of the final manuscript: All authors

## Conflicts of Interest

The authors declare no conflicts of interest and no specific funding sources for this work.

## References

[1] Angelo LC, Vieira ML, Rodrigues SL (2007). Echocardiographic reference values in a sample of asymptomatic adult Brazilian population. Arq Bras Cardiol.

[2] Daimon M, Watanabe H, Abe Y (2008). JAMP Study Investigators. Normal values of echocardiographic parameters in relation to age in a healthy Japanese population: the JAMP study. Circ J.

[3] Yao GH, Deng Y, Liu Y (2015). Echocardiographic Measurements in Normal Chinese Adults (EMINCA) Study Investigators. Echocardiographic measurements in normal chinese adults focusing on cardiac chambers and great arteries: a prospective, nationwide, and multicenter study. J Am Soc Echocardiogr.

[4] Sadeghpour A, Shahrabi M, Bakhshandeh H, Naderi N (2013). Normal echocardiographic values of 368 Iranian healthy subjects. Arch Cardiovasc Image.

[5] Sullere V, Jain D, Sullere S, C. Anthony (2018). Global longitudinal strain. ejection fraction, effort tolerance and normal echocardiography measurements in healthy Indians. Indian Heart J.

[6] Qureshi WT, Leigh JA, Swett K (2016). mComparison of echocardiographic measures in a hispanic/ latino population with the 2005 and 2015 American Society of Echocardiography Reference Limits (The Echocardiographic Study of Latinos). Circ Cardiovasc Imaging.

[7] LaBounty TM, Bach DS, Bossone E, Kolias TJ (2019). Effect of race on echocardiographic measures of cardiac structure and function. Am J Cardiol.

[8] Ryu DR (2016). Normal reference values for doppler echocardiography: influences of ageing, gender and ethnicity. J Cardiovasc Ultrasound.

[9] Asch FM, Miyoshi T, Addetia K, WASE Investigators (2019). Similarities and differences in left ventricular size and function among races and nationalities: results of the world alliance societies of echocardiography normal values study. J Am Soc Echocardiogr.

[10] Morais H, Feijão A, De Victória Pereira S (2021). Values of left ventricular diastolic function assessed by left atrial volumes and Doppler echocardiography in healthy Angolans: Effect of age and gender: A pilot study. J Clin Ultrasound.

[11] Lang RM, Badano LP, Mor-Avi V (2015). Recommendations for cardiac chamber quantification by echocardiography in adults: an update from the American Society of Echocardiography and the European Association of Cardiovascular Imaging. J Am Soc Echocardiogr.

[12] Morais H, Feijão A, de Victória Pereira S (2020). Difficulties and pitfalls in performing speckle-tracking echocardiography to assess left ventricular systolic function”. EC Cardiology.

[13] Kou S, Dulgheru RCaballero (2014). Echocardiographic reference ranges for normal cardiac chamber size: results from the NORRE study. Eur Heart J Cardiovasc Imaging.

[14] Nel S, Nihoyannopoulos P, Libhaber E (2020). Echocardiographic indices of the left and right heart in a normal Black African population. J Am Soc Echocardiogr.

[15] Choi JO, Shin MS, Kim MJ (2015). Normal echocardiographic measurements in a Korean population study: part I. Cardiac chamber and great artery evaluation. J Cardiovasc Ultrasound.

[16] Şafak Ö, Gürsoy OM, Karakoyun S (2019). Normal echocardiographic measurements in a Turkish population: The Healthy Heart ECHO-TR Trial. Anatol J Cardiol.

[17] Akasheva DU, Plokhova EV, Tkacheva ON (2015). Age related left ventricular changes and their association with leukocyte telomere length in healthy people. PLoS One.

[18] Arbab-Zadeh A, Dijk E, Prasad A, Fu (2004). Effect of aging and physical activity on left ventricular compliance. Circulation.

[19] Kuznetsova T, Herbots L, Richart T (2008). Left ventricular strain and strain rate in a general population. Eur Heart J.

[20] Bansal M, Mohan JC, Sengupta SP (2016). Normal echocardiographic measurements in Indian adults: How different are we from the western populations? A pilot study. Indian Heart J.

